# Nanomaterial-based detection of circulating tumor cells and circulating cancer stem cells for cancer immunotherapy

**DOI:** 10.1186/s40580-024-00466-x

**Published:** 2024-12-13

**Authors:** Yeochan Yun, Seewoo Kim, Sang-Nam Lee, Hyeon-Yeol Cho, Jeong-Woo Choi

**Affiliations:** 1 Department of Bio and Fermentation Convergence Technology, Kookmin University, 77 Jeongneung-ro, Seongbuk-gu, Seoul, 02707 Republic of Korea; 2 Department of Chemical and Biomolecular Engineering, Sogang University, 35 Baekbeom-ro, Mapo-gu, Seoul, 04107 Republic of Korea; 3 Uniance Gene Inc., 273, Digital-ro, Guro-gu, Seoul, 08381 Republic of Korea

**Keywords:** Circulating tumor cells (CTCs), Circulating cancer stem cells (CCSCs), Nanomaterial-based detection, Cancer immunotherapy, Personalized cancer treatment

## Abstract

Nanomaterials have emerged as transformative tools for detecting circulating tumor cells (CTCs) and circulating cancer stem cells (CCSCs), significantly enhancing cancer diagnostics and immunotherapy. Nanomaterials, including those composed of gold, magnetic materials, and silica, have enhanced the sensitivity, specificity, and efficiency of isolating these rare cells from blood. These developments are of paramount importance for the early detection of cancer and for providing real-time insights into metastasis and treatment resistance, which are essential for the development of personalized immunotherapies. The combination of nanomaterial-based platforms with phenotyping techniques, such as Raman spectroscopy and microfluidics, enables researchers to enhance immunotherapy protocols targeting specific CTC and CCSC markers. Nanomaterials also facilitate the targeted delivery of immunotherapeutic agents, including immune checkpoint inhibitors and therapeutic antibodies, directly to tumor cells. This synergistic approach has the potential to enhance therapeutic efficacy and mitigate the risk of metastasis and relapse. In conclusion, this review critically examines the use of nanomaterial-driven detection systems for detecting CTCs and CCSCs, their application in immunotherapy, and suggests future directions, highlighting their potential to transform the integration of diagnostics and treatment, thereby paving the way for more precise and personalized cancer therapies.

**Graphical Abstract:**

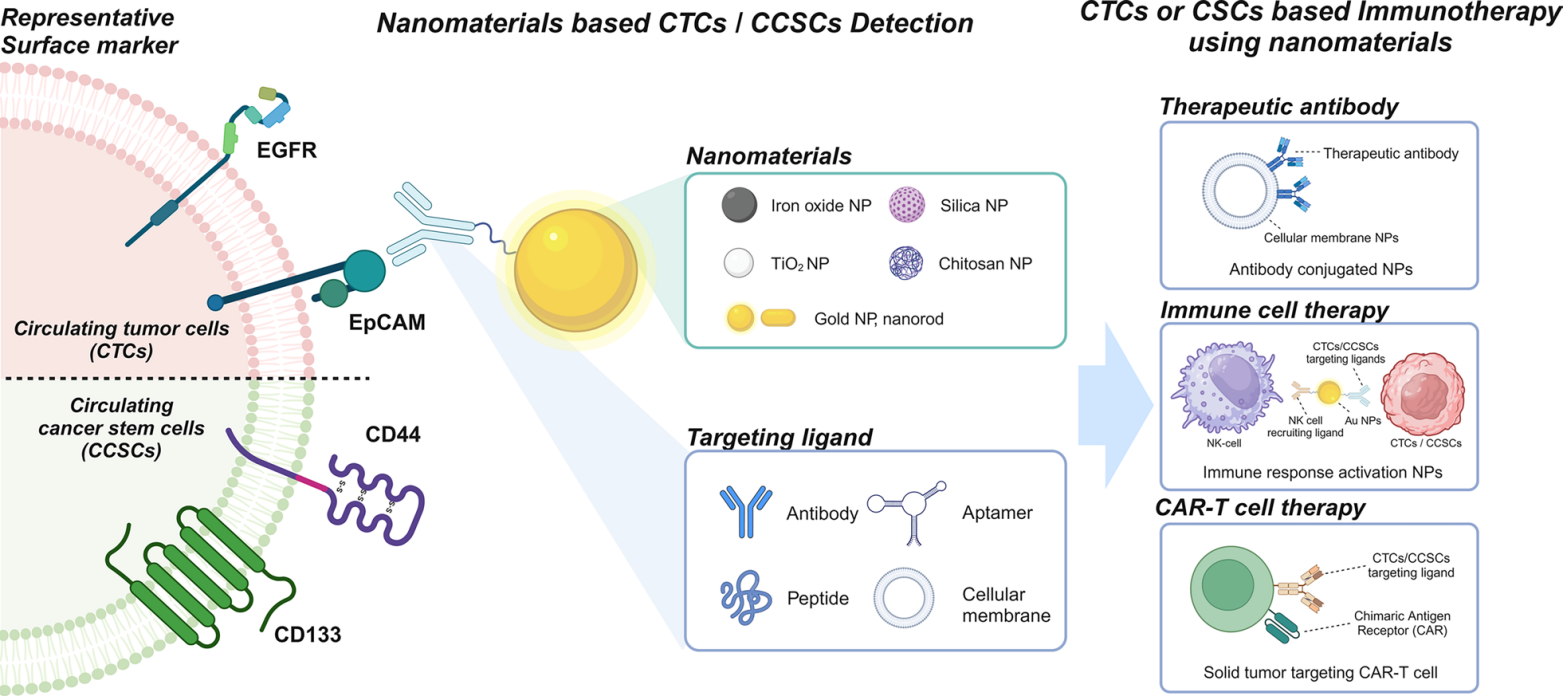

## Introduction

The identification and isolation of circulating tumor cells (CTCs) and circulating cancer stem cells (CCSCs) are critical for understanding cancer metastasis, resistance to treatment, and disease progression [1, 2]. These rare cells, which detach from primary or metastatic tumors and enter the bloodstream, can provide real-time insights into cancer status. Their detection and characterization are particularly important for cancers with high metastatic potential, such as breast, colorectal, pancreatic, and lung cancers [3–6]. Conventional methods for their detection rely on the use of molecular markers that are specific to epithelial or stem-like phenotypes. However, these techniques are limited by low sensitivity or specificity, largely due to the low abundance and heterogeneity of these cells.

Nanomaterials, such as gold, magnetic, and silica-based nanomaterials, have distinct physicochemical properties that can be tailored for the isolation, detection, and analysis of CTCs and CCSCs [7, 8]. These nanomaterials can be functionalized with biomolecules such as antibodies, aptamers, or peptides for target-specific isolation of CTCs or CCSCs from a number of hematopoietic cells in the blood [9–11]. CTCs or CCSCs detection methods such as fluorescence and Raman spectroscopy enable sensitive detection using precisely designed nanomaterials. Furthermore, through the utilization of nanoparticles, cancer immunotherapy applications are being advanced by leveraging their ability to evade immune responses, suppress metastasis, and specifically deliver drugs to tumor sites [12–16].

**Fig. 1:**
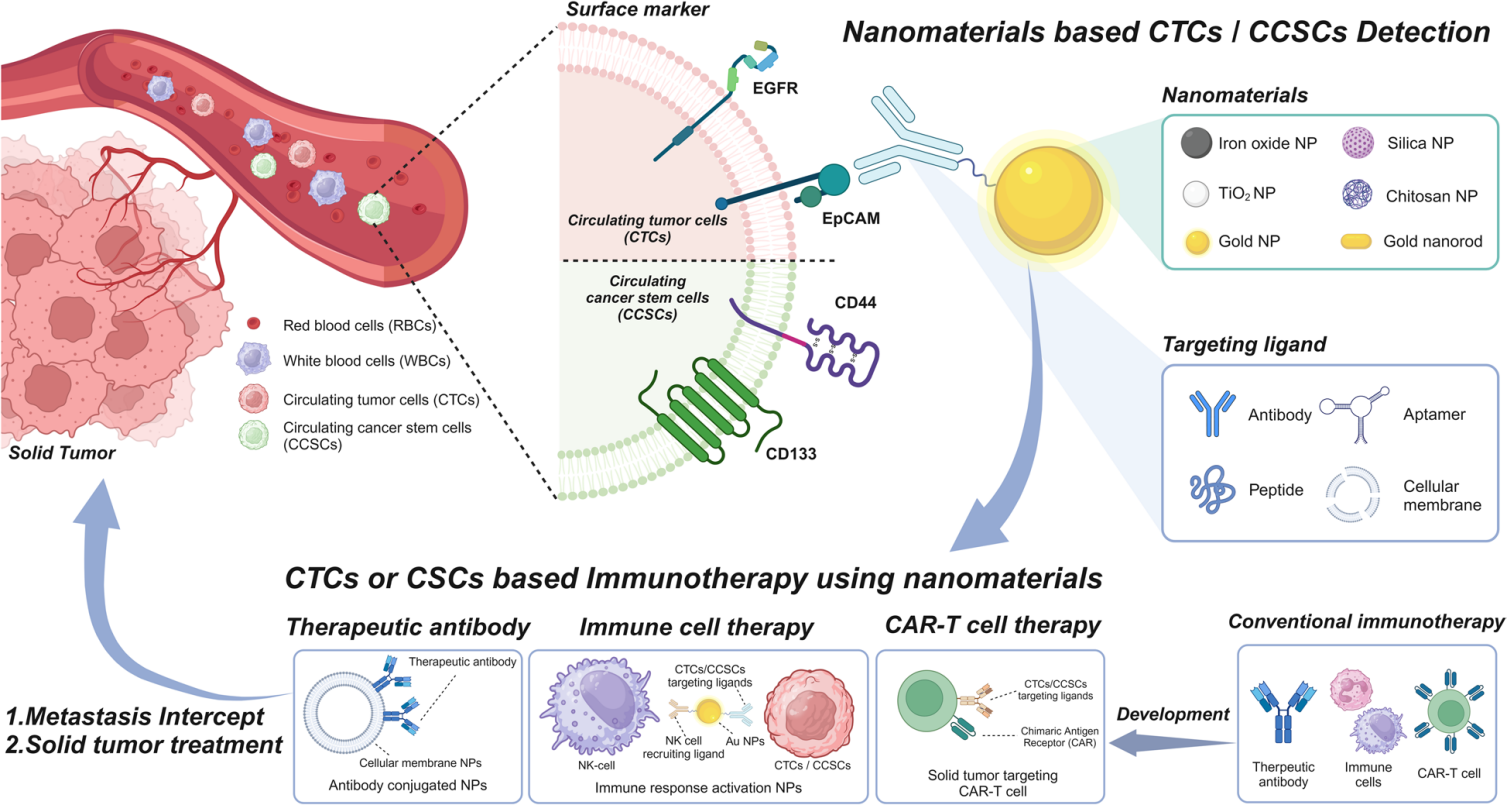
Introduction of nanomaterial-based detection of circulating tumor cells and circulating cancer stem cells for cancer immunotherapy. (Created with BioRender.com)

This review discusses recent advances in nanomaterial-based systems for detecting CTCs and CCSCs, with a focus on their applications in cancer immunotherapy (Fig. 1). First, we will discuss the key molecular markers used in the detection of CTCs and CCSCs, providing detailed insights into how these markers reflect the biological characteristics of various cancer subtypes. Second, we will examine different nanomaterials utilized in detection platforms, such as gold and magnetic nanoparticles (MNPs), and their functionalization for improving cell isolation efficiency. Finally, we will highlight how these nanomaterial-based detection techniques are being applied in cancer immunotherapy, including their role in enhancing the efficacy of immune checkpoint inhibitors and chimeric antigen receptors (CAR)-T cell therapies. This is the first comprehensive review to focus on the utilization of nanomaterials for the detection of CTCs and CCSCs, as well as their application in immunotherapy. We also suggest future directions to refine these technologies further. By integrating nanotechnology with immunotherapy, significant strides can be made toward more precise, personalized cancer treatment strategies. Overall, this review aims to provide essential insights into current advances and new perspectives on integrating nanomaterial-based technologies into cancer diagnosis and immunotherapy.

## Key surface markers and associated subtypes for the detection of CTCs and CCSCs

**Fig. 2:**
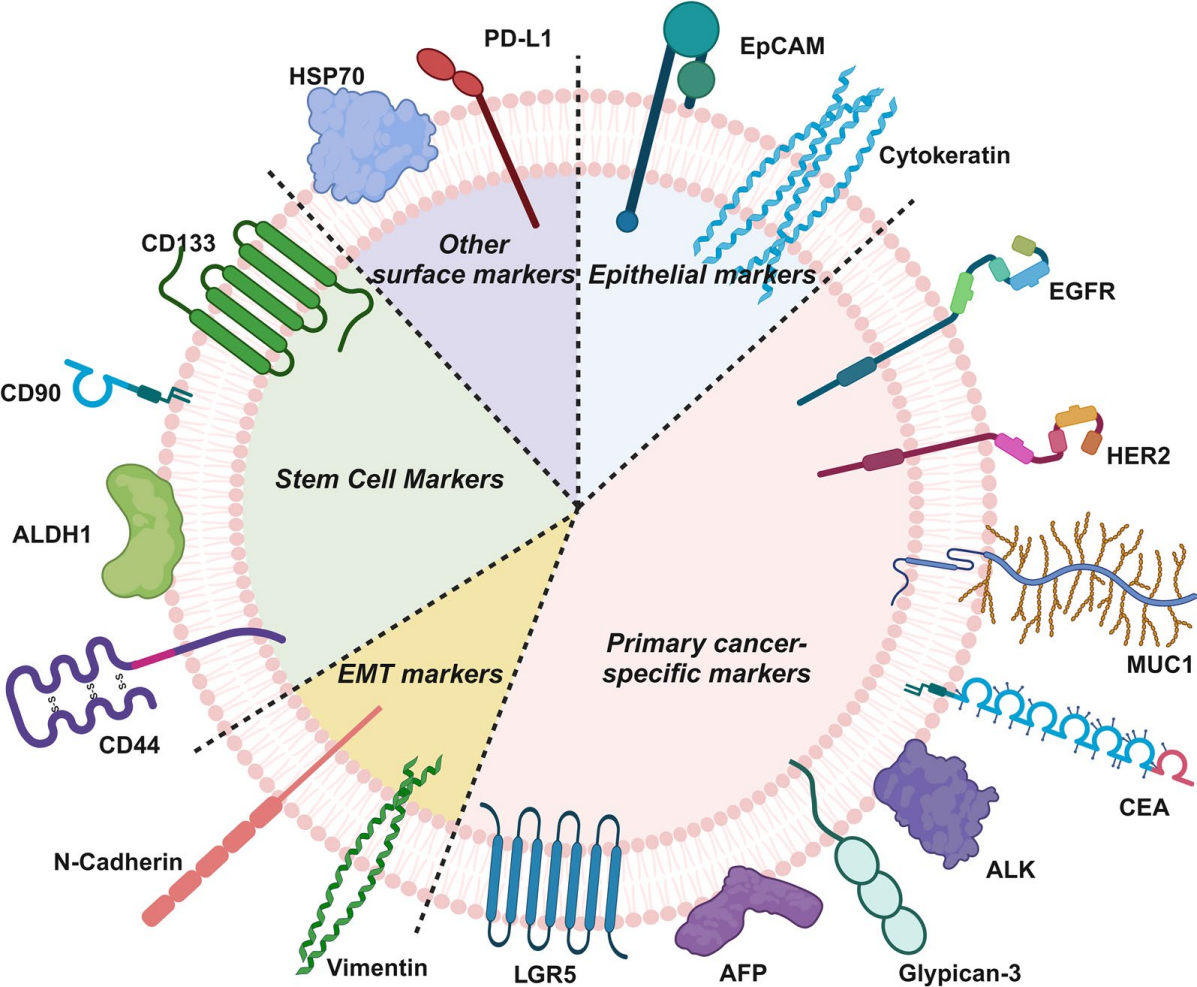
Schematic illustration of key surface markers of CTCs and CCSCs (Created with BioRender.com)

The detection of CTCs and CCSCs relies on specific molecular markers that reflect the unique biological characteristics of primary tumors. This chapter reviews the major surface markers used to detect CTCs and CCSCs, providing detailed information on their characteristics and the cancer subtypes associated with their expression (Fig. 2, Table 1).

**Table 1:** Key surface markers for the detection of CTCs and CCSCs

Primary cancer	Subtype	Surface markers	Refs.
Breast	Luminal-A	EpCAM, CK8, CK18, CK19, MUC1	[19–21, 34–36, 63–65]
	Luminal-B	EpCAM, CK8, CK18, CK19, MUC1, HER2	[19–21, 34–36, 50–53, 63–65]
	HER2-enriched	HER2	[50–53]
	Basal	EGFR	[54–56]
	Triple-negative	ALDH1	[98, 104, 105]
	Breast cancer stem cell	CD44^high^/CD24^low^, ALDH1 Prominin-1 (CD133)	[98, 101, 104, 105, 115–117]
Lung	Non-small cell lung cancer (NSCLC)	EpCAM, cytokeratins, EGFR, ALK, PD-L1	[22, 23, 37–39, 44, 57, 77–80, 121–123]
	Small cell lung cancer (SCLC)	EMT markers (vimentin and N-cadherin)	[92–95]
	Lung cancer stem cell	CD44, CD133, ALDH1	[106, 110]
Liver	Hepatocellular carcinoma (HCC)	EpCAM, AFP, GPC3, CXCR4	[24–26, 84–86, 88–90]
	Liver cancer stem cell	Thy-1 (CD90), CD133, ALDH1	[111–113, 118–120]
Pancreas	Pancreatic ductal adenocarcinoma (PDAC)	EpCAM, CK19, CEA, MUC1	[27–29, 42, 66, 67, 74–76]
	Pancreatic cancer stem cell	CD133, CD44^high^/CD24^high^	[103, 114]
Colon	Adenocarcinoma	EpCAM, CK8, CK18, CK20, CEA, EGFR	[30–32, 43–46, 58–60, 71–73]
	Colorectal cancer stem cell	LGR5, CD133, ALDH1	[81–83, 107–109]

### Epithelial surface markers for CTC detection

Epithelial surface markers of cancer cells are proteins and glycoproteins that reflect the origin and differentiation status of the tumor, playing crucial roles in various physiological processes such as cell–cell interaction, signal transduction, and cell growth and differentiation [17]. These markers are also present in normal epithelial cells but are often overexpressed or altered in cancer cells [18].

#### EpCAM

EpCAM (Epithelial Cell Adhesion Molecule) is a transmembrane glycoprotein involved in cell adhesion, proliferation, and differentiation. It is widely expressed on the surface of epithelial cells and serves as a primary marker for isolating CTCs in most cancers. EpCAM is particularly useful for detecting CTCs in luminal breast cancer (ER^+^/PR^+^) [19–21], non-small cell lung cancer (NSCLC) adenocarcinoma [22, 23], hepatocellular carcinoma (HCC) [24–26], pancreatic ductal adenocarcinoma (PDAC) [27–29], and early-stage colorectal cancer (CRC) characteristics [30–32] where epithelial characteristics are preserved. EpCAM^+^ CTCs in HCC patients indicate a highly aggressive tumor phenotype and may reflect treatment resistance [33]. However, its expression decreases in more advanced tumors due to epithelial-to-mesenchymal transition (EMT).

#### Cytokeratins

Cytokeratins are intracellular filament proteins that contribute to the structural integrity of epithelial cells and serve as markers for CTCs with epithelial origins. CK8, CK18, and CK19 are commonly used to identify CTCs in breast [34–36], lung [37–39], HCC [40, 41], PDAC [27, 42], and epithelial-like CRC subtypes [43], particularly in tumors with epithelial features. CK7 is often expressed in epithelial-like NSCLC [44], while CK20 is widely used to detect CTCs in CRC [44–46].

### Primary cancer-specific surface markers for CTC detection

Primary cancer-specific surface markers are defined as surface proteins that are uniquely expressed or significantly overexpressed in specific types of cancer [47]. These markers serve as important indicators that distinguish cancer cells from normal cells, and as such, they are valuable for cancer diagnosis and targeted therapy [48].

#### Human epidermal growth factor receptor 2 (HER2, for breast cancer)

HER2 is a transmembrane receptor overexpressed in 15–20% of breast cancers [49]. It plays a crucial role in promoting cell proliferation and metastasis [50–53]. HER2-overexpressing CTCs can be detected to monitor the progression of HER2^+^ breast cancer and to assess the responses to anti-HER2 therapies like trastuzumab.

#### Epidermal growth factor receptor (EGFR, for breast, lung, and pancreatic cancer)

EGFR is a transmembrane receptor tyrosine kinase part of the HER family and plays a crucial role in cellular proliferation, survival, and migration. EGFR overexpression is a hallmark of more aggressive cancers, including the basal-like subtype of breast cancer [54–56] and NSCLC adenocarcinomas [57]. This makes EGFR a valuable marker for detecting CTCs yielded by these subtypes, where traditional epithelial markers like EpCAM may be unreliable due to EMT. EGFR^+^ CTCs are also found in a subset of KRAS wild-type CRC patients that may respond well to anti-EGFR therapies [58–60]. EGFR expression is prevalent in non-mucinous adenocarcinomas. The detection of EGFR^+^ CTCs is particularly relevant in guiding targeted therapy decisions [61] and detecting mutations related to resistance to tyrosine kinase inhibitor (TKI) treatment (e.g., T790M) [62].

#### Transmembrane glycoprotein mucin 1 (MUC1, for breast and pancreatic cancer)

MUC1 is a glycoprotein expressed on the surface of epithelial cells, including breast cancer cells [63–65]. It plays a role in cell adhesion and immune evasion. MUC1 is highly expressed in luminal subtypes, particularly luminal A and HER2-enriched breast cancers. The expression of MUC1 in CTCs typically shows a more epithelial-like phenotype. MUC1^+^ CTCs are also commonly associated with PDAC and indicate aggressive and metastatic behaviors [27, 66, 67]. MUC1 is particularly useful for detecting mucinous subtypes of pancreatic cancer, where the overproduction of mucins contributes to tumor progression. The detection of MUC1^+^ CTCs can also indicate poor prognosis and advanced progression of the disease.

#### Carcinoembryonic antigen (CEA, for lung, colorectal, and pancreatic cancer)

CEA^+^ CTCs are most commonly detected in the adenocarcinoma subtypes of NSCLCs [68–70], CRC [71–73] and PDAC [27, 74]. CEA levels in the blood are widely used as a diagnostic and prognostic marker for CRC. Detecting CEA^+^ CTCs provides valuable insights into the status and metastatic potential of CRC and PDAC patients. However, CEA expression is often lower in poorly differentiated or mucinous subtypes of colorectal and pancreatic cancer [75, 76].

#### Anaplastic lymphoma kinase (ALK, for lung cancer)

ALK gene rearrangements are present in a small subset of lung cancers, primarily in NSCLC adenocarcinomas [77, 78]. ALK^+^ lung cancers can be treated with ALK inhibitors [79]. Therefore, detecting ALK expression in CTCs provides insights into therapeutic responses. ALK rearrangements are found in about 3–5% of patients with NSCLC adenocarcinomas [80]. Detecting ALK^+^ CTCs can help monitor patients undergoing ALK-targeted therapies, such as crizotinib. ALK is not typically expressed in SCLCs or squamous cell carcinomas.

#### Leucine-rich repeat-containing G-protein coupled receptor 5 (LGR5, for CRC)

LGR5^+^ CCSCs are commonly detected in primary and metastatic CRCs, particularly in the adenocarcinoma subtypes [81–83]. LGR5 is crucial for identifying stem-like cancer cells that drive tumor initiation and recurrence. The presence of LGR5^+^ CCSCs is associated with a highly aggressive tumor phenotype and poor prognosis, particularly in cases of early-stage CRC, where stem-like cells contribute to tumor development.

#### Alpha-fetoprotein (AFP, for liver cancer)

AFP is a glycoprotein produced by the fetal liver and is often elevated in the serum of HCC patients. AFP is a well-established biomarker used for the diagnosis and prognosis of HCC [84–86]. AFP^+^ CTCs are commonly found in HCC, particularly in the non-progenitor-like subtypes. AFP is often used to monitor tumor burden and recurrence in HCC patients. AFP^+^ CTCs are highly specific to HCC and are associated with advanced disease progression and poor outcomes [87].

#### Glypican-3 (GPC3, for liver cancer)

GPC3 is a heparan sulfate proteoglycan overexpressed in HCC [88–90]. It promotes tumor growth by modulating different signaling pathways. GPC3 is specific to HCC and is not expressed in normal adult liver tissues. GPC3^+^ CTCs are often detected in advanced HCC and indicate more aggressive behavior. GPC3 is frequently expressed in poorly differentiated HCC subtypes, and its expression in CTCs may indicate increased metastatic potential. Targeting GPC3 has emerged as a potential therapeutic strategy for treating advanced HCC, making its detection clinically relevant.

### EMT markers for CTC detection

EMT markers in cancer refer to proteins whose expression changes as cancer cells lose epithelial characteristics and acquire mesenchymal traits [91]. EMT markers are used as important biomarkers to predict the metastatic potential of cancer.

#### Vimentin

Vimentin is an intermediate filament protein that is a hallmark of epithelial-to-mesenchymal transition (EMT), a process that increases cell motility and invasiveness. Vimentin is widely used to detect mesenchymal-like CTCs in aggressive cancer subtypes such as small cell lung cancer (SCLC), advanced NSCLC, and poorly differentiated pancreatic cancer [92, 93]. Vimentin expression in CTCs is associated with increased metastatic potential and poor prognosis in these cancers, making it an important marker for monitoring disease progression.

#### N-cadherin

N-cadherin is a cell adhesion molecule expressed on mesenchymal cells and is another key marker for detecting EMT in CTCs. During EMT, N-cadherin replaces E-cadherin and contributes to increased cell migration and invasiveness. N-cadherin is frequently upregulated in mesenchymal-like CTCs from metastatic CRC and advanced HCC, SCLC, and certain aggressive forms of NSCLC [94, 95]. Its presence in CTCs is indicative of aggressive, therapy-resistant tumors and is associated with poor clinical outcomes.

### Stem cell markers for CCSC detection

Stem cell markers are surface proteins that play a crucial role in identifying a specialized subpopulation of cancer cells with self-renewal and differentiation capabilities [96]. Therefore, stem cell markers serve as important targets for developing therapeutic strategies aimed at cancer stem cells, offering the potential for more effective cancer treatment [97].

#### CD44 and CD24

CD44 and CD24 are important markers for the identification of CCSCs in various tumors [98–100]. CD44 is involved in cell adhesion, migration, and signal transduction, while CD24 plays a role in cell differentiation. The CD44^high^/CD24^low^ phenotype is a hallmark of CCSCs, particularly in basal-like breast cancer [101], NSCLC, particularly in the squamous cell carcinoma variant [102], and PDAC [103]. In these cancers, CD44^high^/CD24^low^ cells exhibit increased metastatic potential, and resistance to chemotherapy, and are associated with poor clinical outcomes.

#### Aldehyde dehydrogenase 1 (ALDH1)

ALDH1 is an enzyme involved in detoxification and cellular metabolism and is a widely recognized marker of cancer stem cells. ALDH1 is expressed in several cancers, including breast [98, 104, 105], lung [106], and CRC [107–109]. ALDH1^+^ CCSCs are associated with increased metastatic potential, therapy resistance, and poor prognosis, particularly in TNBC, advanced NSCLC, and metastatic CRC.

#### CD133 (Prominin-1)

CD133 is a cell surface glycoprotein that serves as a marker for stem cells and CCSCs in several tumor types. Although it is more commonly associated with lung [110], liver [111–113], colorectal [107–109], and pancreatic cancers [103, 114], CD133 has also been detected in a subpopulation of breast cancer stem cells, particularly in more aggressive and treatment-resistant cases [115–117]. CD133 expression is more likely to be associated with the basal-like and TNBC subtypes dominated by stem-like characteristics, leading to increased invasiveness, treatment resistance, and a higher potential for metastasis.

#### Thy-1 (CD90)

CD90 is a glycoprotein involved in cell adhesion and migration. It has emerged as an important marker for liver cancer stem cells (LCSCs) [118–120]. CD90^+^ cells exhibit stem-like properties and are associated with high risks of metastasis and recurrence. CD90 is predominantly expressed LCSCs, which are associated with tumor initiation and treatment resistance. CD90^+^ CTCs and CCSCs are often found in poorly differentiated HCCs and are associated with aggressive behavior, increased metastatic potential, and poor clinical outcomes. CD90 is highly relevant to detecting CCSCs in advanced HCC.

### Other surface markers for CTC and CCSC detection

In addition to epithelial, cancer-specific, EMT, and stem cell markers, there are other important markers used to detect CTCs and CCSCs, particularly in the context of immunotherapy and cellular stress responses. These markers provide critical insight into immune evasion and tumor resistance mechanisms.

#### Programmed death-ligand 1 (PD-L1)

PD-L1 is a transmembrane protein involved in immune evasion that is commonly overexpressed in various cancers, including lung cancer [121–123]. PD-L1 expression is commonly associated with NSCLC, particularly in tumors that are responsive to immunotherapies, such as pembrolizumab and nivolumab. PD-L1 expression in CTCs can reflect the tumor's ability to evade immune detection. SCLC may also express PD-L1 but at a lower level than NSCLC.

#### Membrane-bound HSP70

Membrane-bound HSP70 (heat shock protein 70) is a stress-inducible chaperone protein that is typically found intracellularly but can be translocated to the cell membrane in some cancer cells. Membrane-bound HSP70 is involved in protecting tumor cells from stress-induced apoptosis and promotes immune evasion by inhibiting the cytotoxic activity of immune cells. The presence of membrane-bound HSP70 on CTCs and CCSCs has been reported in several cancer types, including colorectal, pancreatic, and lung cancer. This marker is particularly useful for identifying CTCs that are resistant to stress and may contribute to cancer progression and metastasis.

## Nanomaterials-based isolation and detection of CTCs or CCSCs

Various techniques involving cell surface markers are being utilized to specifically and sensitively isolate and detect CTCs or CCSCs. Among these techniques, those that utilize the unique properties of nanoparticles are particularly effective.

### Nanomaterials

Nanomaterials are utilized across various fields to leverage their unique physicochemical properties such as high surface area at the nanoscale. In addition to these characteristics, nanomaterials showing high biocompatibility and excellent interactions with the cells and tissues of interest are being explored for biological and medical applications. Notably, gold, magnetic, and silica materials are widely used, often in the form of nanoparticles, nanopatterns, or nanowires.

#### Gold nanomaterials

Gold nanomaterials are widely used for biological and medical applications due to their unique physicochemical properties [124–126]. They exhibit high electrical conductivity and stability, and when designed at the nanoscale, their increased surface area enhances their ability to bind efficiently with a wide range of biological and chemical molecules [127–129]. Furthermore, gold nanoparticles can absorb or scatter light at specific wavelengths, making them ideal for applications like fluorescence imaging and Raman spectroscopy [130–132]. Their high biocompatibility allows their administration into the body, making them an excellent material for the isolation and detection of CTCs or CCSCs [133].

**Fig. 3:**
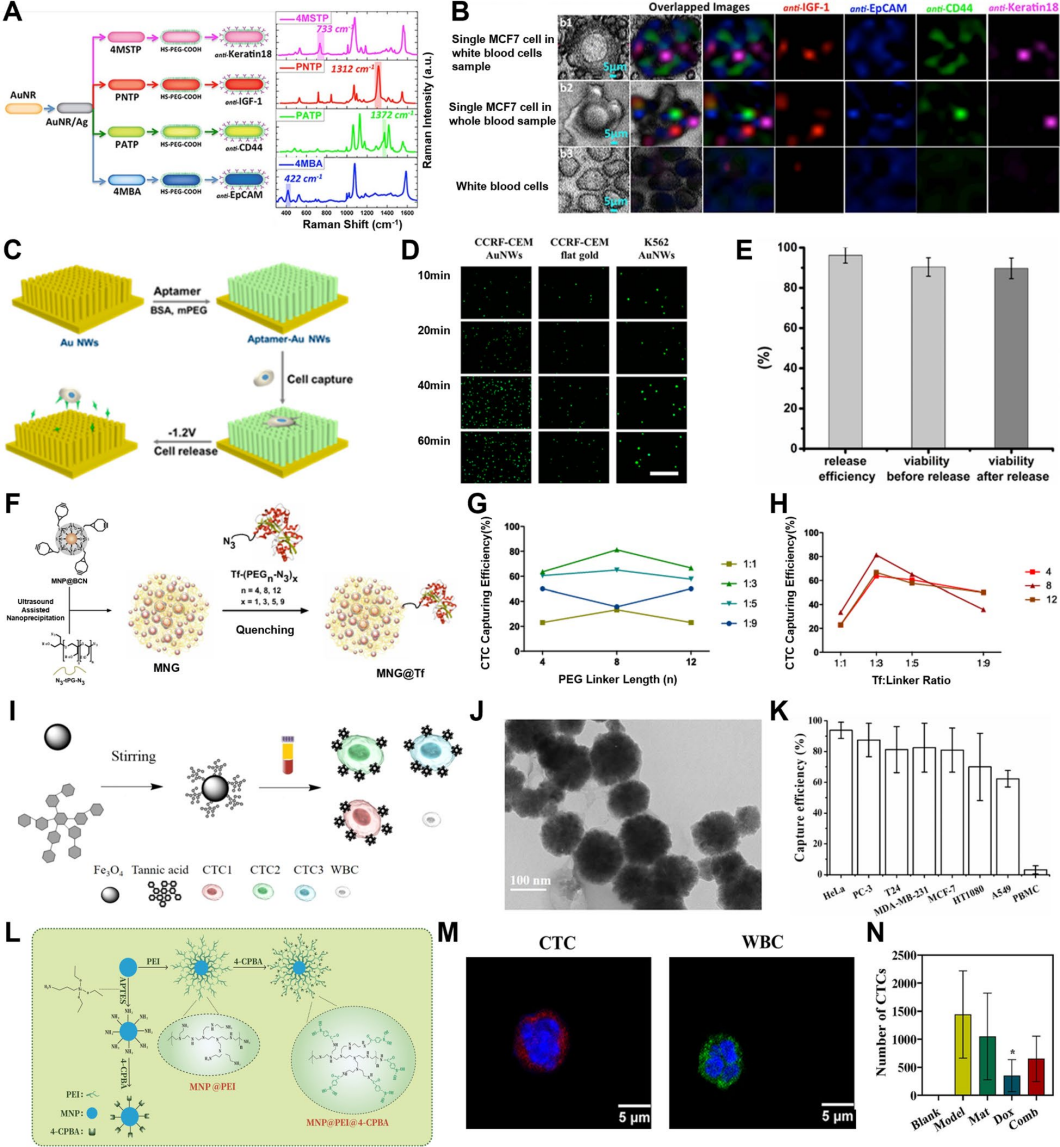
CTCs or CCSCs isolation and detection with gold nanomaterials and magnetic nanoparticles (MNPs). **A** Schematic illustration of Raman spectra for the four families of SERS nano-agents. **B** Multicolor SERS analysis of MCF-7 cells in blood. (Reproduced with permission from ref. [134]. Copyright 2014, The Authors) **C** Schematic illustration of the AuNWs for CTC capture and release. **D** Fluorescent microscope images of captured cells at each incubation time. **E** Change of release efficiency and cell viability during the releasing process. (Reproduced with permission from ref. [135]. Copyright 2017, American Chemical Society) **F** Schematic illustration of MNGs@Tf synthesis by ultrasound-assisted nanoprecipitation. **G** CTC (HCT 116) capturing efficiency represented against the linker length. **H** CTC (HCT 116) capturing efficiency represented against the Tf to PEG linker ratio. (Reproduced with permission from ref. [146]. Copyright 2018, The Authors) **I** Schematic illustration of TA-functionalized magnetic nanoparticles (MNPs-TA) to isolate CTCs of different subtypes from blood samples. **J** TEM image of MNPs-TA for characterization **K** Capture yields of MNPs-TA for different types of cancer cells. (Reproduced with permission from ref. [147]. Copyright 2021, American Chemical Society.) **L** Schematic illustration of PBA-modified MNPs. **M** Fluorescence images of captured cells in tumor-bearing mice blood samples (Blue: Nuclei, Green: CD45, Red: PanCK). **N** The number of isolated CTCs from the blood of all mice. (Reproduced with permission from ref. [148]. Copyright 2024, Elsevier B.V.)

Utilizing these properties, a highly sensitive method for CTC detection has been developed based on silver-coated gold nanorods [134]. The nanorods were conjugated with antibodies targeting various cancer cell markers, such as EpCAM, CD44, Keratin 18, and IGF-1 receptor, to specifically bind and detect MCF-7 cells (Fig. 3A). The combination of surface-enhanced Raman scattering (SERS) and photothermal resonance enabled multiplexed detection of CTCs directly from blood samples. Furthermore, a cocktail of four distinct SERS nano-agents enabled enhanced spectral differentiation of the target cells, resulting in highly accurate CTC detection in blood samples (Fig. 3B).

Additionally, gold nanowires (AuNWs) have been explored for efficient isolation of CTCs and their electrochemical release [135]. These AuNWs were fabricated through electrochemical deposition on a template of anodic aluminum oxide (AAO) and were functionalized with aptamers to enable binding to specific cells (Fig. 3C). Their high surface area enabled binding of a large number of aptamers, significantly improving cell isolation efficiency compared to AuNWs not functionalized with aptamers (Fig. 3D). To detach cells from the AuNW array without causing damage, a negative potential of -1.2 V was applied for 30 s, performed as electrochemical desorption. As a result, the thiol-gold bonds between the aptamers and AuNWs were broken, releasing the captured CTCs with a viability of 90% (Fig. 3E). This demonstrates the utility of AuNWs for CTC isolation in diagnostic and therapeutic applications.

Moreover, a method of labeling CTCs with gold nanoparticles for Inductively Coupled Plasma-Mass Spectrometry (ICP/MS)-based detection has been explored [136]. Using anti-EpCAM antibodies immobilized on a microplate, HepG2 cells could be detected. Here, streptavidin was used as a bridge for asialoglycoprotein receptor (ASGPR), which is known to be specific to hepatocytes, where rolling circle amplification (RCA) primers were attached for DNA amplification. The gold nanoparticles were attached with a complementary DNA capable of detecting the amplified DNA, resulting in a DNA bundle densely coated with gold nanoparticles. This approach enabled sensitive detection of rare amounts of CTCs, with a detection limit as low as 3 CTCs (15 cells/mL).

#### Magnetic nanomaterials

MNPs can be classified into metallic and metal oxide types depending on the type of constituent elements and are sometimes synthesized by doping with additional elements or alloying to have strong magnetization ability [137]. MNPs are excellent materials for isolating specific cells from blood because they can be easily manipulated by an external magnetic field due to their strong magnetic properties [138–140]. Additionally, magnetic nanomaterials, such as nanopatterns, nanowires, and nanogels containing magnetic nanoparticles, can leverage the magnetic properties of these nanoparticles to facilitate the detection of CTCs or CCSCs more effectively [141, 142]. This capability has been explored for the isolation and analysis of CTCs or CCSCs to facilitate early diagnosis and treatment monitoring of cancer [143–145]. For example, magnetic nanogels (MNGs) have been employed for CTC detection [146]. These MNGs were functionalized with transferrin (Tf) via polyethylene glycol (PEG) linkers, which allowed selective capture of CTCs with overexpressed Tf receptors (Fig. 3F). The research team tested various lengths of PEG linkers and Tf-to-linker conjugation ratios for optimization of CTC capture efficiency. Specifically, when the PEG linker consisted of 8 ethylene glycol units and at the Tf-to-linker conjugation ratio of 1:3 (Fig. 3G), the capture efficiency in human blood samples reached its peak at 81% (Fig. 3H).

Additionally, tannic acid, a water-soluble polyphenolic compound containing multiple phenolic hydroxyl groups, has been shown to effectively interact with the glycocalyx of cancer cells to mediate efficient isolation of CTCs [147]. Specifically, the MNPs surface-functionalized with tannic acid (Fig. 3I, J) effectively inhibited nonspecific adhesion to peripheral blood mononuclear cells while efficiently isolating CTCs in the blood. The isolation efficiencies for seven different types of cancer cells, including HeLa (cervical cancer), PC-3 (prostate cancer), T24 (bladder cancer), MDA-MB-231 (triple-negative breast cancer), MCF-7 (luminal A breast cancer), HT1080 (fibrosarcoma), and A549 (lung adenocarcinoma), ranged from 62.3 to 93.7% (Fig. 3K).

Furthermore, MNPs functionalized with dendritic boronic acid have demonstrated high specificity and sensitivity in detecting CTCs from the blood in a mouse model of metastatic breast cancer while avoiding the capture of white blood cells [148] (Fig. 3L). These nanoparticles successfully identified CTCs capable of being metastasized in the lung. Immunostaining with CD45 and Pan-CK antibodies confirmed the identity of captured cells as CTCs (Fig. 3M). Furthermore, in a mouse tumor model treated with chemotherapeutics, the detection of CTCs in the blood was significantly reduced, confirming the validity of the approach (Fig. 3N).

#### Silica nanomaterials

Silica-based nanomaterials are widely used in various biological applications due to their excellent chemical stability and biocompatibility [149, 150]. In addition, silica-based nanomaterials can be easily transformed into various forms, so they can be utilized as nanoparticles, nanowires, etc. [151, 152]. In CTC detection platforms, the use of nanoparticles or nanostructures increases the efficiency of capturing CTCs by enhancing interactions with cells through a large surface area. Furthermore, they can easily be combined with optical detection techniques and microfluidic devices [153–155], allowing the detection and quantification of isolated CTCs at enhanced sensitivity and specificity. This makes them capable of simultaneous detection of multiple biomarkers.

**Fig. 4:**
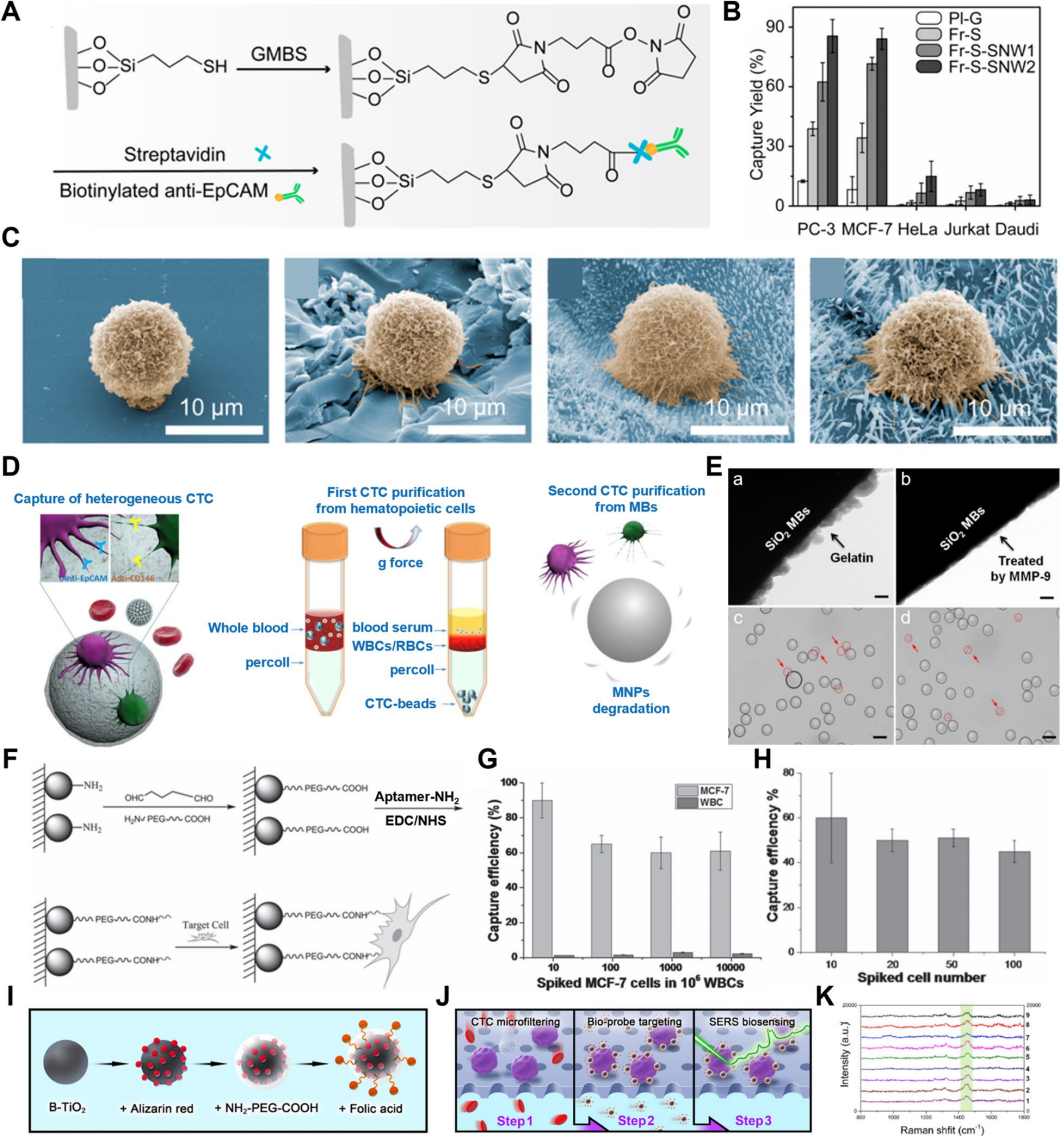
CTCs or CCSCs isolation and detection with silica and other nanomaterials. **A** Schematic illustration of silica nanowires (SNWs) functionalization with EpCAM antibody. **B** Capture specificity between EpCAM positive cell lines (PC-3, MCF-7) and EpCAM negative cell lines (HeLa, Jurkat, and Daudi) (Pl-G: plain glass slides. Fr-S: frosted slides) **C** SEM images of Interactions of cancer cells and interfacial structures (Pl-G, Fr-S, Fr-S-SNW1, Fr-S-SNW2). (Reproduced with permission from ref. [156]. Copyright 2018, American Chemical Society) **D** Schematic illustration of CTC isolation and release protocol using multifunctional silica microbeads. **E** TEM images of (a) a gelatin nanoparticle-coated silica microbead (SiO2@Gel MB) and (b) a SiO2@Gel MB after gelatin degradation induced by the MMP-9 enzyme. (c-d) Microscopic images of specifically captured MCF-7 cells purified from microbeads after MMP-9 treatment. (Ea, Eb Scale bar: 50 nm; Ec, Ed, Scale bar: 50 μm). (Reproduced with permission from ref. [157]. Copyright 2018, Ivyspring International Publisher) **F** Schematic illustration of bio-conjugating PEG and DNA-aptamer onto chitosan particles (CNPs). **G** Capture efficiency between spiked MCF-7 cells and WBCs under different spiked MCF-7 cell numbers. **H** Capture efficiency of MCF-7 cells in blood samples on CNPs. (Reproduced with permission from ref. [160]. Copyright 2015, John Wiley and Sons) **I** Schematic illustration of the synthesis process of SERS bio-probe. J. Schematic illustration of CTC detection steps. **K** SERS spectral of the captured cancer cells. (Reproduced with permission from ref. [163]. Copyright 2022, Elsevier B.V.)

In a previous study, silica nanowires were grown on commercially available frosted glass surfaces to engineer a large surface area needed for CTC detection [156]. Anti-EpCAM antibodies were attached to the surface of these silica nanowires to enhance the capture efficiency of EpCAM^+^ cells (Fig. 4A). Specifically, this biochip demonstrated a high capture efficiency of ~ 85.4% for prostate cancer cells (PC-3), which was better than that of a conventional flat glass slide (Fig. 4B). This high efficiency was attributed to the synergistic effects of the micro-concave and convex structures of the frosted glass combined with the nanostructure of silica nanowires. It was confirmed that the interaction between nanostructures and cell filopodia plays an important role in capturing CTCs, as the capture efficiency increased with longer nanowires (Fig. 4C).

In another study, silica microbeads coated with gelatin nanoparticles functionalized with anti-EpCAM and anti-CD146 antibodies (SiO2@Gel MBs) were used to effectively capture cancer cells that had undergone EMT and exhibited reduced EpCAM expression [157] (Fig. 4D). Density gradient centrifugation was employed to isolate high-purity CTCs from blood samples, and the gelatin coating was degraded using the MMP-9 enzyme to release the captured CTCs without inducing damage (Fig. 4E). The CTC capture efficiency of this system exceeded 80%, with a purity of over 85% and viability of 92.5%. These promising results indicated their potential to be used for various downstream analyses, such as genetic profiling or drug sensitivity testing.

#### Other nanomaterials

In addition to the aforementioned nanomaterials, various other nanomaterials have been utilized for the detection of CTCs or CCSCs [158, 159]. For example, biocompatible and biodegradable chitosan nanoparticles have been functionalized with a DNA aptamer and PEG via electrospray deposition [160] (Fig. 4F). This system’s CTC isolation efficiency exceeded 90% in artificial leukocyte samples (Fig. 4G). Furthermore, in the whole blood, the system achieved a CTC isolation efficiency of 45–60% (Fig. 4H).

Additionally, titanium dioxide nanoparticles (TiO₂ NPs), which are widely used in various biological applications due to their photocatalytic properties and chemical stability, have been demonstrated to enhance the sensitivity and accuracy of CTC and CCSC detection by amplifying optical and electrical signals [161, 162]. Black TiO₂ NPs were used to form SERS bio-probes by attaching the Raman reporter molecule alizarin red and PEG-folic acid (FA) onto their surface [163] (Fig. 4I). FA enabled selective binding to folate receptors on the surface of CTCs, thereby avoiding the capture of white blood cells. CTCs were initially separated via microfiltration and subsequently targeted using the bio-probe, enabling CTC detection from patient blood samples through Raman biosensing (Fig. 4J, K).

### Targeting ligands for nanomaterial-based CTCs or CCSCs detection

As discussed above, nanomaterials can be surface-conjugated with various targeting ligands to enhance their target specificity and efficiency. Antibodies, aptamers, peptides, and cell membranes have been explored for the detection of CTCs or CCSCs to specifically recognize or capture unique surface markers. In this section, we introduce various biomaterials used for specific detection of CTC or CCSC.

#### Antibody-conjugated nanomaterials for CTC or CCSC detection

Antibodies are proteins the immune system produces that recognize and bind to antigens, playing a crucial role in defending against foreign substances. Each antibody interacts with specific antigens through unique antigen-binding sites. For the isolation and detection of CTCs or CCSCs, antibodies are functionalized onto the surfaces of detection materials to selectively bind to specific antigens expressed in CTCs or CCSCs, thereby facilitating cell-specific isolation [164–167].

**Fig. 5:**
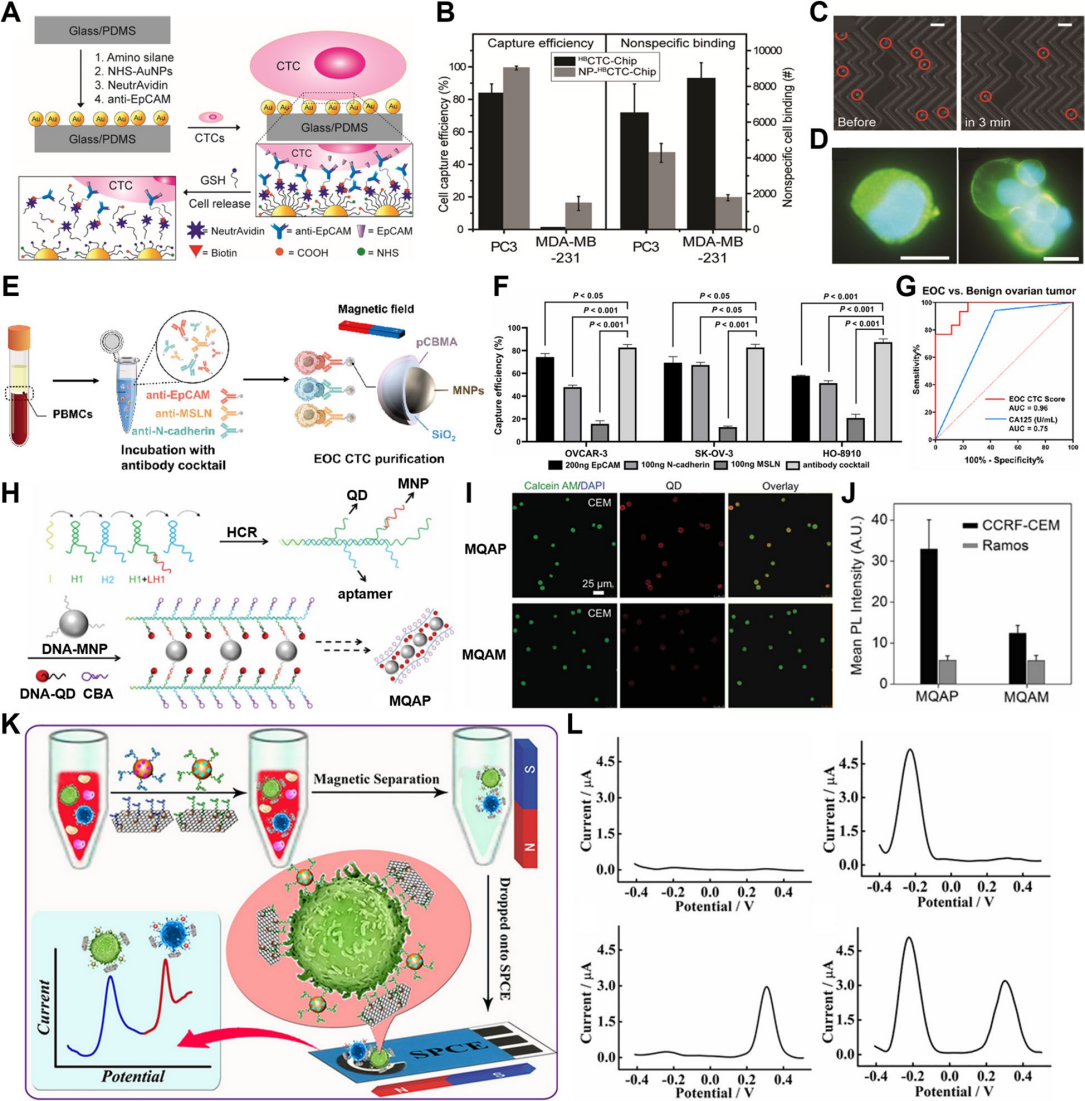
CTCs or CCSCs isolation and detection by antibody and aptamer-conjugated nanomaterials. **A** Schematic illustration of each surface modification process step. **B** Comparison of capture efficiencies and nonspecific binding of cancer cells, PC3 and MDA-MB-231 cells. **C** Bright-field microscopy images of isolated CTCs (Scale bar: 150 μm). **D** Immunofluorescence staining of cell-surface receptors of a captured single CTC and CTC cluster from a metastatic breast cancer patient. (Blue: Nuclear, Green: EpCAM/CDH11, Scale bar: 10 μm). (Reproduced with permission from ref. [170]. Copyright 2017, American Chemical Society) **E** Schematic illustration of multi antibody-modified MNPs for epithelial ovarian cancer (EOC) CTC purification. **F** Capture efficiencies of OVCAR-3, SK-OV-3, and HO-8910 cells with individual and multiple antibodies. **G** Receiver operating characteristic curve (ROC) curves of EOC CTC Scores and serum CA125. (Reproduced with permission from ref. [171]. Copyright 2023, American Chemical Society) **H** Schematic illustration of magnetic nanoparticle-quantum dot (QD)-aptamer copolymers (MQAPs) for magnetic isolation of CTCs. I. Confocal microscopy images of MQAP and MQAM-treated CEM cells. **J** Mean QD PL intensity of CEM cells and Ramos cells treated with MQAPs and MQAMs. (Reproduced with permission from ref. [174]. Copyright 2018, John Wiley and Sons) **K** Schematic illustration of capture, isolation, and amplified and multiplexed detection of the target CTCs in whole blood. **L** Square wave voltammetry (SWV) responses of the mixture of the capture probes and signal probes incubated with different target CTCs. (Reproduced with permission from ref. [175]. Copyright 2019, American Chemical Society)

EpCAM is the most widely utilized cancer surface marker [168, 169]. Anti-EpCAM antibodies have been conjugated with biotin and subsequently attached to neutravidin-coated gold nanoparticles on a substrate to facilitate the isolation of CTCs. Here, the captured CTCs could be released by glutathione (GSH) treatment [170]. The chip functionalized with this antibody-conjugated gold nanoparticles demonstrated enhanced capture efficiency for both PC3 and MDA-MB-231 cells compared to the unfunctionalized chip while minimizing nonspecific cell binding (Fig. 5A, B). Furthermore, it was noted that CTCs began to be released after 3 min of GSH treatment (Fig. 5C), where both individual CTCs and CTC clusters could be identified in patient samples through fluorescence staining (Fig. 5D).

Furthermore, multiple target antibodies, including EpCAM, N-cadherin, and mesothelin (MSLN), have been employed to modify the surface of MNPs to capture various subpopulations of CTCs [171] (Fig. 5E, F). For example, to facilitate accurate diagnosis and dynamic monitoring of epithelial ovarian cancer (EOC), multi-antibody-modified MNPs were utilized to isolate CTCs, followed by RNA analysis. These isolated CTCs were examined for nine EOC-specific genes using droplet digital PCR (ddPCR), achieving an area under the curve (AUC) value of 0.96. This indicated the high sensitivity and specificity of the approach in differentiating between patients with benign ovarian tumors and those with ovarian cancer (Fig. 5G). This strategy has the potential to be applied in cancer diagnosis and post-treatment monitoring of EOC patients.

#### Aptamer-conjugated nanomaterials for CTC or CCSC detection

Aptamers are short, single-stranded DNA or RNA oligonucleotide sequences that can bind with high affinity and specificity to particular molecules or cells. Their function is similar to that of antibodies by binding with antigens, but they offer the advantages of smaller size and easier synthesis. When functionalized on nanoparticles, aptamers can detect markers expressed in CTCs or CCSCs [9, 172, 173].

By utilizing the DNA hybridization properties of aptamers, the sgc8c DNA aptamer, which specifically recognizes protein tyrosine kinase 7 (PTK7) overexpressed in CCRF-CEM leukemia cells, has been used along with DNA-quantum dots (QDs) and DNA-MNPs to form MNP QD-aptamer monomers (MQAMs) [174] (Fig. 5H). When multiple MQAMs were polymerized to form MQA polymers (MQAPs), the QD signal enhanced, and a higher capture efficiency was achieved during magnetic isolation (Fig. 5I). Furthermore, the sgc8c aptamer exhibited specific binding to PTK7, resulting in a detectable signal from CEM cells but not from Ramos cells (Fig. 5J).

A method for detecting CTCs using multiple aptamers has also been investigated. For example, gold nanoparticles have been arrayed onto magnetic graphene sheets, with both Sgc8 and Td05 aptamers attached to facilitate the detection of CCRF-CEM and Ramos cells [175] (Fig. 5K). Each cell type could be magnetically separated from the whole blood, and CTC detection was confirmed by measuring the square wave voltammetry (SWV) response using disposable screen-printed carbon electrodes (SPCEs) (Fig. 5L).

#### Peptide-conjugated nanomaterials for CTC or CCSC detection

Peptides are chain-like structures with amino acid linkages. Due to their structural diversity and biological activity, they play a crucial role in various biological recognition and signaling processes. For the detection of CTCs or CCSCs, peptides can function as specialized ligands by binding to specific surface markers associated with cancer [176–178].

**Fig. 6:**
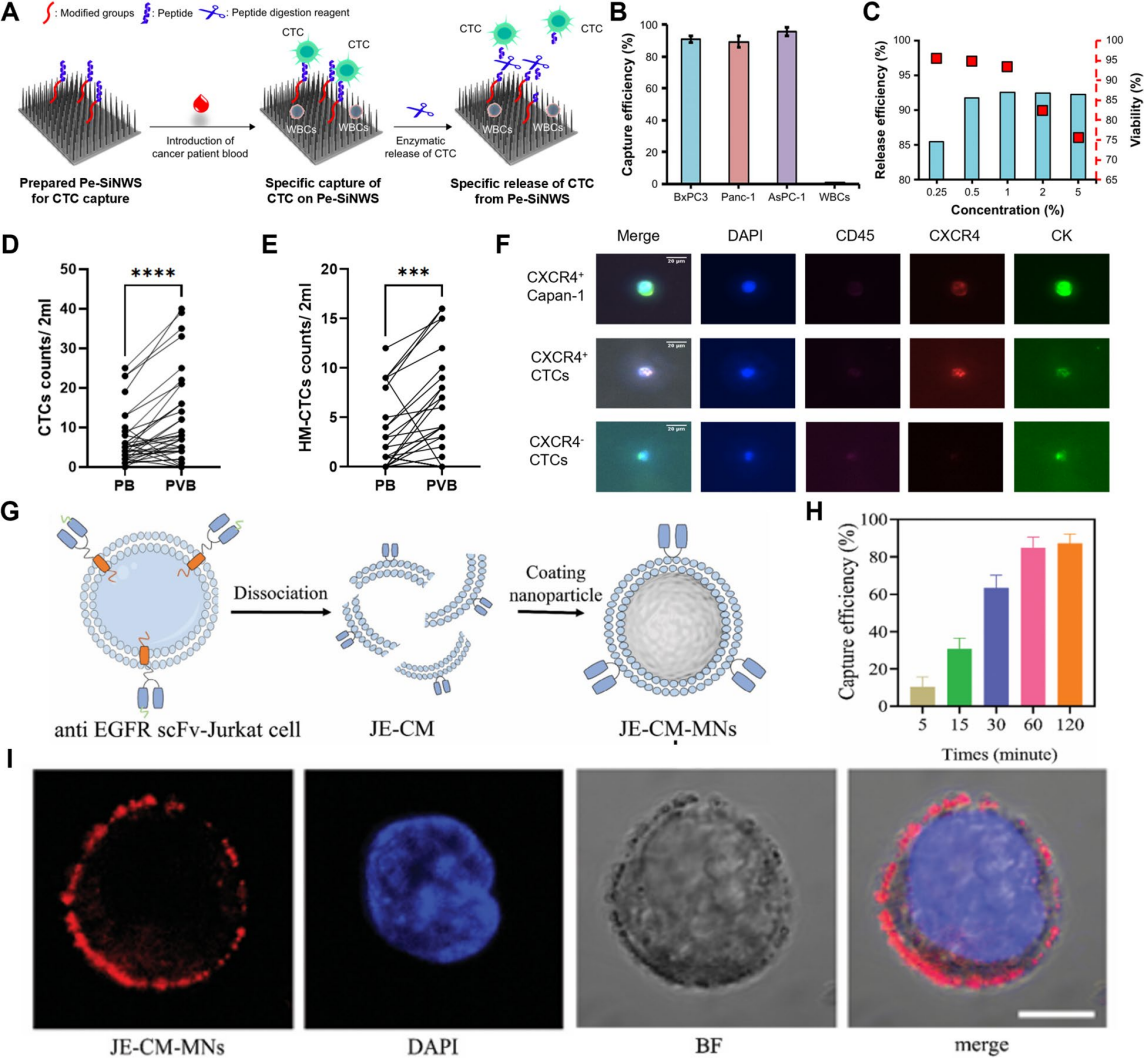
CTCs or CCSCs isolation and detection by peptide and cell membrane-conjugated nanomaterials. **A** Schematic illustration of CTC capture on Pe-SiNWs and release from Pe-SiNWs. **B** Capture efficiency and comparison of different cell lines of pancreatic cancer and WBCs. **C** Effect of different concentrations of chymotrypsin on CTC release efficiency and CTC viability. (Reproduced with permission from ref. [179]. Copyright 2019, The Authors) **D** CTCs counts showed significant differences between PB and PVB in the surgical group. **E** HM-CTCs counts showed significant differences between PB and PVB in the surgical group. **F** Immunofluorescence of captured Capan-1 cells and CXCR4 + /CXCR4- CTCs. (Reproduced with permission from ref. [180]. Copyright 2024, The Authors) G. Schematic illustration of the fabrication of JE-CM-MNs. **H** Capture efficiency of MDA-MB-468 cells at different incubation times with JE-CM-MNs. **I** Confocal images of MDA-MB-468 cells captured by JE-CM-MNs (Scale bar: 10 μm). (Reproduced with permission from ref. [186]. Copyright 2023, John Wiley and Sons)

For example, the CKAAKN polypeptide, which specifically binds to CTCs from pancreatic cancer patients, has been utilized in combination with anti-EpCAM antibodies on silica nanowires [179] (Fig. 6A), facilitating the detection of EpCAM^+^ CTCs as well as EpCAM^−^ CTCs. This approach led to the specific capture of pancreatic cancer cells (Fig. 6B), which could be released through treatment with varying concentrations of chymotrypsin (Fig. 6C).

In another study, Pep10, an EpCAM-targeted peptide, was conjugated to MNPs to synthesize Pep@MNPs for CTC detection [180]. Pep@MNPs showed higher capture efficiency compared to the conventional CellSearch system, with detection efficiencies of 63.3% and 77.5% in the peripheral blood (PB) and portal vein blood (PVB) of pancreatic cancer patients, respectively. Notably, only 2 mL of blood sample was required for analysis (Fig. 6D, E). The identity of the captured CXCR4^+^ CTCs (HM-CTCs) was confirmed through CXCR4, CK, and CD45 immunostaining (Fig. 6F).

#### Cell membrane-conjugated nanomaterials for CTC or CCSC detection

Cell membranes contain various receptors and ligands. Nanomaterials functionalized with cell membranes can utilize these components to facilitate specific interactions with the target cells [181–184]. This approach enables nanoparticles to mimic the characteristics of the cell, the phospholipid bilayer naturally interacts with the biological environment, enhancing stability, and is recognized as self by the body, thereby increasing delivery efficiency.

Nanoparticles known as Neu-IMNs have been developed by functionalizing the surface of MNPs with neutrophil membranes, which are recognized for their ability to interact with CTCs [185]. In systemic circulation, Neu-IMNs effectively avoided the formation of cell clusters, thereby enhancing the detection and isolation of CTCs. These nanoparticles demonstrated a capture efficiency of 96.82% and an isolation purity of 90.68%. They showed selective targeting of CTCs while avoiding WBCs, and the captured CTCs were confirmed through CD45 and CK immunostaining.

**Fig. 7:**
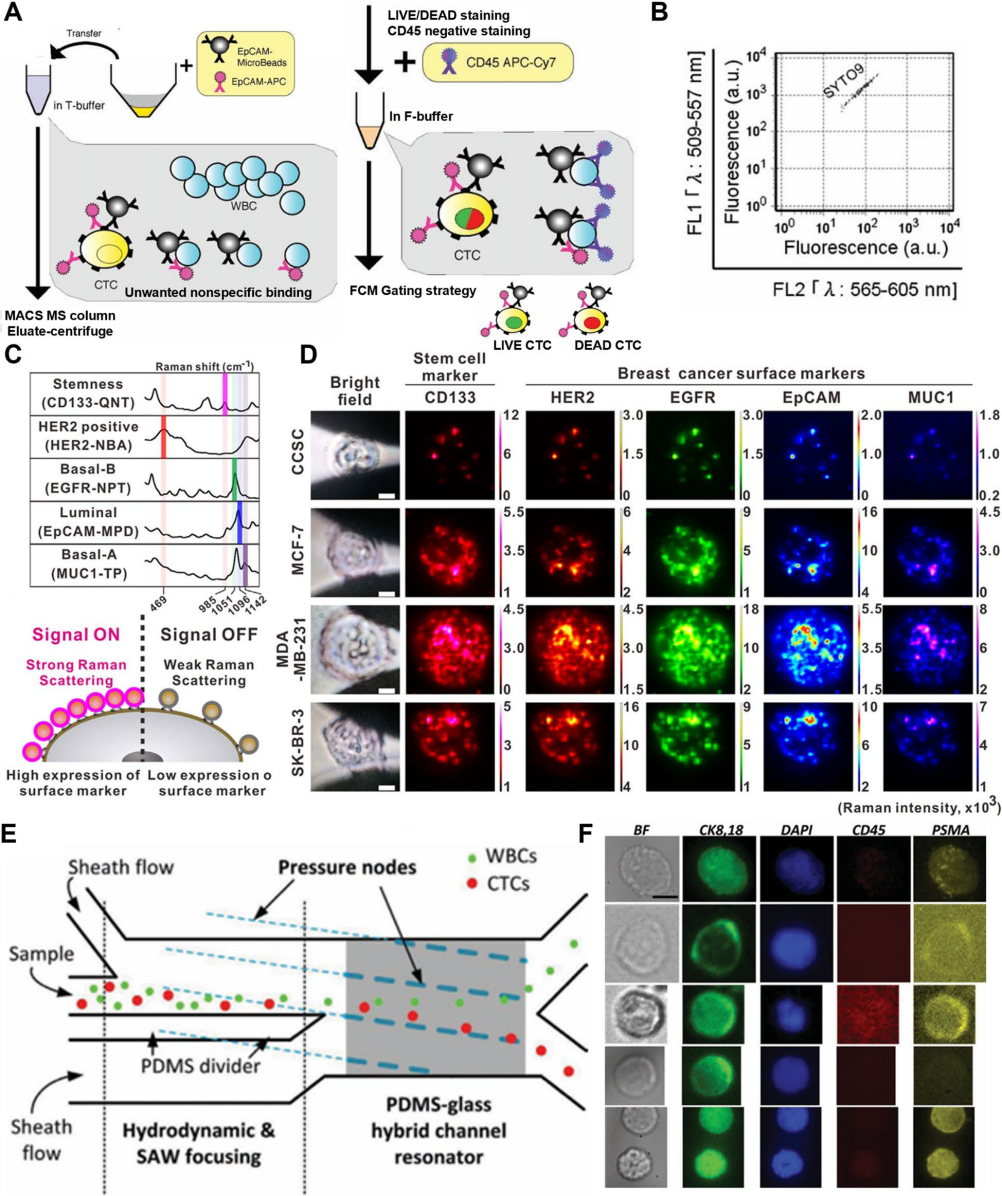
CTCs or CCSCs phenotyping methods. **A** Schematic illustration of the intact CTC enrichment procedure by flow cytometry. **B** Flow cytometry analysis using CD326 APC and LIVE/DEAD (SYTO9 and PI) for extraction and enumeration of live CTC. (Reproduced with permission from ref. [197]. Copyright 2011, John Wiley and Sons) **C** SERS peaks of five different types of RANs as barcoding system and schematic illustration selective encoding of live CCSCs/CTCs by RANs. **D** Optical microscope images and Raman maps obtained from detected CCSCs/CTCs. (Reproduced with permission from ref. [198]. Copyright 2018, Elsevier B.V.) **E** Schematic illustration of the microchannel as viewed from the top. **F** Fluorescence images of the immunostaining pattern for individual CTCs (Scale bar: 10 µm). (Reproduced with permission from ref. [199]. Copyright 2018, John Wiley and Sons)

Additionally, genetic engineering based on lentivirus has been used to facilitate the expression of anti-EGFR scFv and mCherry in the membrane of Jurkat cells [186] (Fig. 7G). By coating these engineered cell membranes onto MNPs, CTCs could be isolated, with the capture efficiency peaking at 60 min post-treatment (Fig. 6H). The mCherry-emitted fluorescence from these nanoparticles could be used for the detection of EGFR^+^ cells (Fig. 6I).

### CTCs or CCSCs phenotyping with nanomaterials

Early detection and diagnosis of cancer hinges on the ability to detect and confirm the presence of rare CTCs or CCSCs in the bloodstream [187]. This information is vital for monitoring disease progression and implementing tailored treatment strategies for different cancers [188]. Common surface markers utilized in cancer detection include EpCAM, HER2, CD44, and EGFR. The analytical techniques employed to detect these markers include flow cytometry [189–191], Raman spectroscopy [192, 193], and immunostaining [194–196].

Phenotyping through flow cytometry enables simultaneous assessment of multiple biological characteristics and surface markers of CTCs. Furthermore, large numbers of pooled cells can be analyzed, which is advantageous for detecting rare CTCs or CCSCs. To utilize flow cytometry for phenotyping analysis, CTCs have been labeled with EpCAM magnetic microbeads for sorting, in which anti-CD45 antibodies were to minimize the interference from white blood cells [197] (Fig. 7A). By performing LIVE/DEAD staining, only cells labeled with SYTO9 could be detected in flow cytometry, enabling the characterization and enumeration of CTCs (Fig. 7B).

The method of analyzing phenotypes based on Raman spectroscopy enables real-time analysis of the biochemical components of cells with high sensitivity and specificity, leveraging distinctive spectral features, low background noise, and signal amplification through SERS. This approach utilizes specific chemical bonds or unique molecular vibrations. For example, a nanoparticle system called the Raman-active nanoprobes (RANs) has been reported [198]. This gold nanoparticle-based system was functionalized with Raman reporters and antibodies that recognize surface markers, enabling simultaneous separation and phenotyping within a microfluidic device. The Raman signal was shown to be dependent on the cell expression levels of surface marker (Fig. 7C). This enabled the identification of cell phenotypes through breast cancer cell markers, such as HER2, EGFR, and EpCAM, as well as the stem cell marker CD133 (Fig. 7D). These nanoparticles successfully identified different CTC and CCSC subtypes from the blood in a xenograft mouse tumor model, thereby confirming their association with metastasis.

Immunostaining utilizes antibodies that bind to specific proteins or antigens to identify the cells of interest. By immunostaining the isolated cells, the phenotypes of CTCs and CCSCs can be analyzed using optical techniques. For example, in a study where acoustic and microfluidic technologies were integrated to enable the isolation of CTCs, immunostaining was conducted to confirm the absence of a membrane protein specific to prostate cells [199] (Fig. 7E). Furthermore, the origins of various cancer cell types could be identified through immunostaining of unconventional surface markers (Fig. 7F).

## Applications in cancer immunotherapy based on CTCs and CCSCs detection using nanomaterials

The detection of CTCs and CCSCs using the aforementioned nanoparticles is being explored for various applications, including cancer metastasis prediction, cancer treatment, and disease progression monitoring. This section introduces various methods for integrating these technologies into cancer diagnosis and immunotherapy [200, 201].

### Cancer immunotherapy based on CTCs and CCSCs detection

As previously outlined, the analysis of the phenotype of CTCs or CCSCs can be employed as a means of implementing immunotherapy [202, 203]. Immunotherapy represents a treatment method that leverages the body's immune system to directly target the tumor and its surrounding microenvironment [204–206]. This approach entails either the activation of immune cells or the direct utilization of immune cells for the recognition and treatment of cancer cells [207, 208].

The clinical use of immunotherapy includes therapeutic antibodies, CAR-T cells, and immune checkpoint inhibitors. One strategy is the use of therapeutic antibodies, which are designed to target specific antigens on cancer cells for destruction by the immune system. An alternative approach is CAR-T cell therapy, which involves the engineering of T cells to express CARs that target specific tumor antigens, thereby enhancing the targeting of cancer cells [209]. Furthermore, immune checkpoint inhibitors are drugs that have been developed to block proteins that inhibit T-cell activation, thereby enhancing the body's immune response against cancer [210]. These therapeutic modalities have been demonstrated to be effective in inducing tumor-specific immune responses, eliminating cancer cells, and preventing cancer recurrence.

Particularly in immunotherapy, the utilization of CTCs enables their application by assessing PD-L1 expression, among other markers. For instance, PD-L1 expression on CTCs provides critical real-time insights into patient responses to PD-1/PD-L1 immune checkpoint inhibitors [211]. Patients with high PD-L1 expression on CTCs have demonstrated better disease control rates when treated with PD-1 inhibitors. Furthermore, tracking changes in PD-L1 expression levels on CTCs during treatment allows for the evaluation of therapeutic efficacy and the prediction of progression-free survival (PFS), making it a non-invasive approach to guide immunotherapy [212].

### Immunotherapy based on CTCs and CCSCs detection with nanomaterials

CTCs or CCSCs spread to other organs through the bloodstream during the metastatic process. These cells evade immune surveillance and proliferate, which underscores the importance of controlling cancer progression and preventing recurrence [213]. Immunotherapy offers a range of strategies to overcome the immune evasion mechanisms of CTCs and CCSCs and apply effective immunotherapeutic approaches (Table 2) [214].

**Table 2:** Advantages of CTCs or CCSCs based immunotherapy using nanomaterials in recent studies

Immunotherapy methods			Description	Advantages	Refs.
Therapeutic antibodies			Combines therapeutic antibodies with nanomaterials to enhance antitumor immune responses	- Improved targeting precision to cancer cells - Enhanced immune system recognition of tumors - Increased therapeutic efficacy in controlling metastasis	[217–219]
Immune cell therapy	Lymphocytes-based therapy	CAR-T cells	Integrates nanomaterials to enhance CAR-T cell specificity and antitumor efficacy	- Increased specificity of CAR-T cells to tumor-related markers - Improved suppression of metastasis - Potential for overcoming immune evasion mechanisms	[226]
		NK cells	Enhances NK cell targeting tumor sites with nanomaterials while preserving their cytotoxic functions to improve cancer immunotherapy	- Improved targeting efficiency of immune cells to tumor sites - Preservation of NK cell cytotoxicity and cytokine production - Improved suppression of tumor growth and metastasis through localized immune activity	[223]
	Neutrophils-based therapy		Enhances neutrophils-based therapy using nanomaterials to optimize efficiency of drug delivery and metastasis prevention	- Selective binding to CTCs - Specific homing to tumor-associated microenvironments - Prevention of metastasis by inhibiting CTC colonization	[224]

#### Therapeutic antibody with nanomaterials

Therapeutic antibodies are monoclonal antibodies that can be used as treatments targeting specific antigens. Current monoclonal antibodies used in cancer therapy independently enhance immune system recognition and destruction, act as immune checkpoint inhibitors to boost immune responses or block proteins that promote cancer cell growth and metastasis [215, 216].

**Fig. 8:**
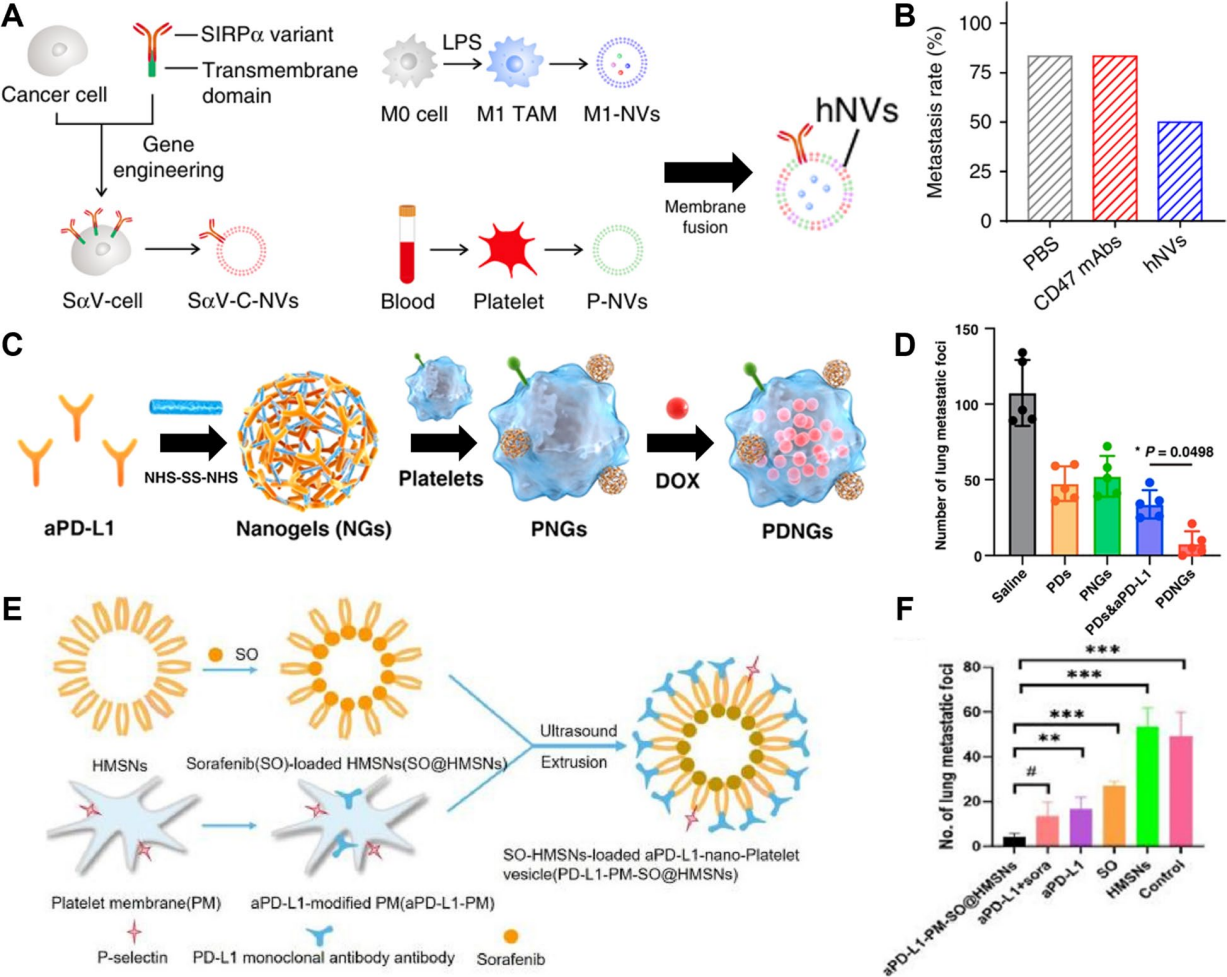
CTCs or CCSCs-based therapeutic antibody treatment with nanomaterials. **A** Schematic illustration of the hybrid cell membrane nanovesicles (hNVs) consisting of engineered SαV-C-NVs, M1-NVs, and P-NVs. **B** Metastasis rates after different treatment groups. (Reproduced with permission from ref. [217]. Copyright 2020, The Authors) **C** Schematic illustration of bioengineered platelets (PDNGs) synthesis. **D** Number of lung metastatic sites after different treatment groups. (Reproduced with permission from ref. [218]. Copyright 2022, American Chemical Society) **E** Schematic illustration of preparation route to aPD-L1-PM-SO@HMSNs. **F** The number of lung metastasis after treatment in different groups. (Reproduced with permission from ref. [219]. Copyright 2023, Elsevier)

For example, hybrid nanovesicles (hNVs) were developed to block the CD47 signal on cancer cells using anti-CD47 antibodies, allowing macrophages to remove cancer cells via phagocytosis [217] (Fig. 8A). hNVs were shown to effectively amplify the antitumor immune response of macrophages and T cells within the tumor microenvironment, as well as inhibit tumor recurrence and distant metastasis. Specifically, hNVs prevented cancer cells from evading the immune system by blocking the CD47-SIRPα signaling axis with anti-CD47 antibodies. These hNVs, combining antibodies and nanomaterials, demonstrated high efficacy in cancer treatment, reducing the tumor recurrence rate to 33% (2 out of 6 mice showed tumor recurrence), and metastasis suppression as an immunotherapy approach, resulting in markedly fewer metastatic foci compared to other treatment groups (Fig. 8B).

Furthermore, Qi Lu et al. used bioengineered platelets (PDNGs) to deliver both anti-PD-L1 antibodies (aPD-L1) and doxorubicin (DOX) to tumor sites [218] (Fig. 8C). The aPD-L1 antibody plays a role in reversing the immunosuppressive tumor microenvironment and activating T cell-mediated immune responses to eliminate cancer cells, while DOX acts as a chemotherapy agent to directly attack tumor cells. PDNGs showed superior performance in capturing and eliminating residual tumor cells and CTCs after surgery, effectively inhibiting metastasis and recurrence (Fig. 8D). Treatment using PDNGs significantly extended survival in a mouse model, with the median survival time increasing by 40% compared to the control group, and markedly reduced tumor recurrence and metastasis rates.

Additionally, CTCs form a protective shield by binding with platelets in the bloodstream, which hinders the efficacy of drugs on CTCs. To address this, a study utilized platelet membrane-coated nanoparticles to exploit the interaction between CTCs and platelets, thereby enabling the precise delivery of the immune checkpoint inhibitor PD-L1 monoclonal antibody and the anticancer drug sorafenib, which effectively inhibited tumor growth [219] (Fig. 8E). This system demonstrated high accumulation at tumor sites, enhanced immune responses, and strengthened the ability of T cells to attack cancer cells (Fig. 8F). The study showed that platelet-functionalized nanoparticles have a significant potential for combination therapy involving anticancer drugs and immunotherapy.

#### Immune cell therapy with nanomaterials

Immune cell therapy involves utilizing or engineering immune cells for the treatment of cancer and other diseases. Immune cells, including T cells, NK cells, and dendritic cells, are employed to directly target cancer cells or to stimulate immune responses [220, 221]. Recently, technologies have emerged that involve conjugating immune cell membranes to nanoparticles to enhance their delivery to tumor sites, thereby maximizing interactions with cancer cells and improving therapeutic efficacy [222]. The combination of immune cell therapy and nanomaterials offers advantages by enhancing the targeting efficiency of immune cells, boosting the immune response, and effectively suppressing metastasis and recurrence.

**Fig. 9:**
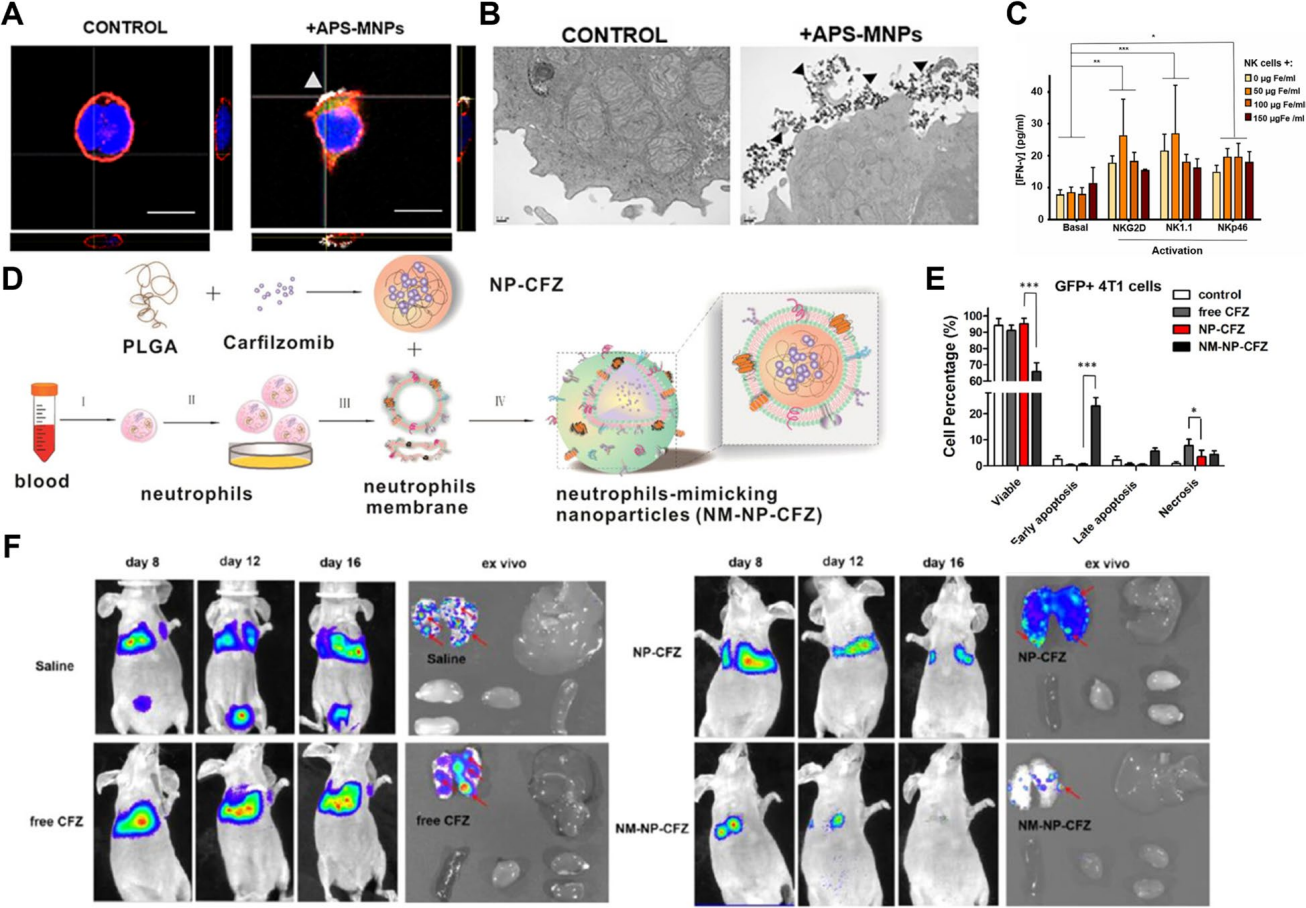
CTCs or CCSCs-based immune cell therapy with nanomaterials. **A** Confocal images of the NK-92MI cells after treatment with APS-MNPs (Grey: NPMs, Blue: nucleus, Red: cell membrane, Scale bar: 10 μm). **B** TEM images of NK-92MI cells after treatment with MNPs. **C** Quantification of IFN-γ production by murine NK cells. (Reproduced with permission from ref. [223]. Copyright 2019, The Authors) **D**. Schematic illustration of neutrophil-mimicking nanoparticles (NM-NP-CFZ) synthesis. **E** Percentage of viable, early apoptotic, late apoptotic, and necrotic cells among GFP + 4T1 cells. **F** BLI imaging of mice treated with each condition. (Reproduced with permission from ref. [224]. Copyright 2017, American Chemical Society)

In a direct application of immune cell-based therapy, MNPs were used in adoptive cell transfer therapy to more effectively guide NK cells to tumor sites, aiming to improve their tumor-targeting efficiency while maintaining their functional activity [223]. NK cells play a crucial role in tumor immunity. To concentrate them at tumor sites, MNPs were attached to the NK cells' surface and guided to the target area using an external magnetic field (EMF) (Fig. 9A, B). The modified NK cells retained their primary functions, including cell-killing ability, interferon-gamma (IFN-γ) production, and cell surface marker expression (Fig. 9C). This study demonstrated the potential for enhancing cancer treatment by combining nanotechnology with immune cell-based therapy.

The binding capacity of neutrophil cell membranes to CTCs and cells in inflammatory environments is attributed to the presence of various membrane proteins [224]. Ting Kang et al. coated biodegradable nanoparticles with neutrophil membranes, developing neutrophil-mimicking nanoparticles (NM-NPs) that effectively capture CTCs and bind to inflammatory cells, thereby inhibiting the spread of metastatic cancer cells (Fig. 9D). Notably, NM-NPs demonstrated robust adhesion to CTCs while they were in circulation in the blood (Fig. 9E). This prevented cancer cells from establishing themselves in metastatic sites. Furthermore, NM-NPs loaded with the anticancer drug carfilzomib effectively inhibited CTC survival and metastasis, as well as suppress the growth of cancer cells at established metastatic sites (Fig. 9F). This demonstrates that nanomaterials utilizing cell membranes can be used as an efficacious therapy to block CTCs and metastasis at an early stage when combined with immunotherapy.

The role of platelets in protecting cancer cells during CTC formation and aiding in immune evasion makes it crucial to disrupt the interactions between platelets and CTCs to inhibit tumor metastasis. The development of synthetic nanoplateletsomes, which fuse platelet membranes with synthetic lipid membranes to prevent binding to CTCs and inhibit metastatic tumor formation, represents a promising avenue of research [225]. In a mouse preclinical model, these nanoplateletsomes effectively inhibited CTC survival, reducing CTC counts by approximately 90%, and suppressed the growth of metastatic tumors, with metastatic nodules in the lungs either completely absent or significantly fewer in treated mice compared to controls, indicating a potential for further investigation.

Additionally, research has been conducted using CAR-T cell therapy to target markers directly associated with CTCs to reduce their numbers and suppress cancer metastasis [226]. The HSP70 protein, which is typically located in the cytoplasm, relocates to the cell membrane by binding to phosphatidylserine or globotriaosylceramide (Gb3) lipids in cancer cells. GrB-CAR T cells, engineered to express granzyme B (GrB), a natural ligand targeting HSP70, were employed to suppress metastasis driven by CTCs expressing mHSP70. The expression of mHSP70 in cancer cells was effectively suppressed by GrB-CAR T cells. In an NCG mice xenograft model, in which AsPC-1 and SK-Hep-1 cells were used, the targeting of metastatic CTC clusters with GrB-CAR T cells resulted in a greater reduction in CTCs and inhibition of lung tumor metastasis compared to the use of CD19-CAR T cells.

## Conclusions

The integration of nanomaterials has resulted in a revolutionary advancement in the detection of CTCs and CCSCs, significantly impacting the field of cancer diagnostics and therapeutic monitoring, particularly in immunotherapy. The employment of gold, magnetic, and silica-based nanoparticles has resulted in a notable enhancement in the sensitivity and specificity of CTC and CCSC isolation from blood samples, which is of paramount importance for the early detection of cancer and the formulation of personalized treatment strategies. Notably, these developments extend beyond the domain of diagnostics, offering direct avenues for enhancing immunotherapy through the translation of CTC and CCSC detection platforms into therapeutic applications.

Nanomaterial-based systems, which are adept at isolating and phenotyping CTCs and CCSCs, provide valuable real-time feedback on tumor dynamics, metastasis, and treatment resistance. This capability has a direct impact on the development of immune-based therapies, including immune checkpoint inhibitors and CAR-T cells. By linking nanomaterials with phenotypic techniques such as Raman spectroscopy and microfluidics, researchers can refine immunotherapy protocols, targeting specific CTC or CCSC markers to enhance therapeutic efficacy.

One of the most significant advantages of nanomaterials in immunotherapy is their capacity to convey therapeutic agents, such as monoclonal antibodies, directly to CTCs and CCSCs. This targeted delivery enhances the immune system's ability to recognize and destroy tumor cells, thereby offering new possibilities in combating immune evasion mechanisms employed by CTCs. The combination of CTC detection technologies with immunotherapy offers a promising approach to reducing relapse and metastasis, particularly in the context of aggressive cancers.

Future research should focus on the integration of nanomaterials into CAR-T cell therapy, whereby the detection technologies currently utilized for CTCs can be adapted to enhance CAR-T cell targeting. Nanomaterials can facilitate the engineering of CAR-T cells that selectively target CTC markers like EpCAM or HER2, thereby overcoming barriers in solid tumors [227, 228]. Moreover, nanomaterials can be utilized to facilitate the delivery and persistence of CAR-T cells or the recruitment of NK cells within the immunosuppressive tumor microenvironment, thereby improving therapeutic outcomes [229]. Additionally, if nanoparticles are applied to CAR-Macrophage therapy [230, 231], as in CAR-T cell therapy, their unique properties could be utilized to overcome immune evasion mechanisms and enhance specificity, potentially achieving greater therapeutic efficacy.

In conclusion, the cross-application of nanomaterials-based CTC and CCSC detection technologies to immunotherapy has the potential to be a transformative advancement in cancer treatment. These innovations are positioned to facilitate convergence between diagnostic and therapeutic modalities, thereby enabling more precise and personalized strategies for combating cancer.

## Data Availability

Not applicable.
